# Editorial: Microbial Electrogenesis, Microbial Electrosynthesis, and Electro-bioremediation

**DOI:** 10.3389/fmicb.2021.742479

**Published:** 2021-09-13

**Authors:** Sebastià Puig, Ludovic Jourdin, Shafeer Kalathil

**Affiliations:** ^1^LEQUiA, Institute of the Environment, University of Girona, Girona, Spain; ^2^Department of Biotechnology, Faculty of Applied Sciences, Delft University of Technology, Delft, Netherlands; ^3^Hub for Biotechnology in the Built Environment, Department of Applied Sciences, Faculty of Health and Life Sciences, Northumbria University, Newcastle, United Kingdom

**Keywords:** microbial electrochemical technologies, 3-D printing, electro microbiology, synthetic electrodes, kinetics modelling

Microbial electrochemical technology (MET) is a bioelectrochemical platform with the potential to overcome a broad range of environmental issues (i.e., global energy, climate crises and water scarcity, among others). This emerging field of research represents an interdisciplinary approach that combines the strengths of microbial catalysis, synthetic electrodes, and analytical techniques for the production of energy and chemicals from waste carbon. The interest in METs has been growing over the years. As an example, the number of papers published in 2020 for the keywords of this Research Topic were ‘Microbial Electrogenesis (10 papers; 9 in 2017)’, ‘Microbial Electrosynthesis (83 papers; 61 in 2017)’, and ‘Electro-bioremediation (51 papers; 25 in 2017)’ according to the Scopus database (July 29th, 2021).

Of particular interest are electroactive microbes with the naturally-evolved ability to electrically interact with insoluble electron acceptors or donors (metal oxides/electrodes) for anaerobic respiration or fermentation, which promises broad applications in microbial fuel cells (Lovley and Holmes, 2021), microbial electrosynthesis (MES; Dessì et al., 2021) and electro-bioremediation (Wang et al., 2020). A key limitation of METs arises from the imperfect integration of microbes with electrode materials which limits the power output, treatment capacity, or volumetric productivity depending on the application (Domínguez-Garay et al., 2018; Fang et al., 2020; Jourdin et al., 2020). Therefore, the nexus of breakthroughs lie in the electrode architecture and microbial interfaces.

The articles published in this Research Topic represent a step forward in the development of METs. For instance, Erben et al. studied the influence of local acidification on the current production of *Shewanella oneidensis* MR-1/electrode composites using various buffer concentrations from 10 to 40 mM. The results suggest that proton transport associated with electron transfer dictates the rate of extracellular electron transport in *S. oneidensis* MR-1 in agreement with a previous report (Okamoto et al., 2017). This study concluded that current production was dictated by the chemical (micro)-environment (i.e., local acidification) and the anode material to promote biofilm growth. The electrospun anode materials proposed in this study could provide a solution for high current densities by facilitating biofilm formation while minimising media costs.

MES systems are plagued with poor-performing electrodes that limit microbial activity (Prévoteau et al., 2020; Dessì et al., 2021; Jourdin and Burdyny, 2021). Significant efforts have been given to develop improved cathodes for MES. Kracke et al. propose the use of 3D-printed custom electrodes to fine-tune H_2_ delivery during MES. The idea behind this is to integrate a 3D fabricated carbon aerogel cathode plated with nickel-molybdenum and *Methanococcus maripaludis* for electromethanogenesis. Specifically, the nickel-molybdenum coating catalysed the H_2_ evolution reaction catalysts that provided H_2_ to *M. maripaludis* while the 3D structure enhanced the catalytically active surface area. These modifications could mitigate the effects of bubble formation and local pH gradients within the boundary layer, hence overcoming some key constraints on *in situ* electron delivery in MES.

Breakthrough understanding of the process limiting steps in MES can be achieved using computational modelling tools. Cabau-Peinado et al. developed a general framework for modelling microbial kinetics in biofilm-driven MES of carboxylates (e.g., acetate, n-butyrate, and n-caproate) from CO_2_. The model was fitted and validated using experimental data from different research groups. The results indicate significant substrate limitation (as CO_2_ dissolved concentration) in existing MES systems and suggest that operating MES in continuous mode (as constant CO_2_ sparging and continuous flow of fresh medium) enhances microbial growth and allows higher current densities to be achieved.

Another interesting approach is the use of electro fermentation to overcome the metabolic limitations of fermentative pathways. Application of current can alter metabolic pathways in fermentative microbes and can yield energetically unfavourable products which are not commonly produced by traditional fermentation reactions (Schievano et al., 2016; Logan et al., 2019). Isipato and colleagues proposed electro fermentation to enhance propionate production via controlling lactate fermentation and to recycle the resulting CO_2_ into acetate, thus increasing the volatile fatty acid yield and reducing the addition of chemicals for pH control (Isipato et al.).

In summary, we believe that the collection of articles included in this Research Topic can pave the way for the development of a high-performing bioelectrochemical platform for sustainable power generation and chemical synthesis.

## Author Contributions

All authors listed have made a substantial, direct and intellectual contribution to the work, and approved it for publication.

## Funding

SP is a Serra Húnter Fellow (UdG-AG-575) and acknowledges the funding from the ICREA Acadèmia award and the Spanish Ministry of Science (RTI2018-098360-B-I00). LEQUIA (http://www.lequia.udg.edu/) has been recognized as a consolidated research group by the Catalan Government (2017-SGR-1552). LJ acknowledges the funding from Shell and a PPP-allowance from Top Consortia for Knowledge and Innovation (TKI's), of the Ministry of Economic Affairs and Climate in the context of the TU Delft e-Refinery Institute. SK acknowledges Research England's Expanding Excellence in England (E3) Fund.

## Conflict of Interest

The authors declare that the research was conducted in the absence of any commercial or financial relationships that could be construed as a potential conflict of interest.

## Publisher's Note

All claims expressed in this article are solely those of the authors and do not necessarily represent those of their affiliated organizations, or those of the publisher, the editors and the reviewers. Any product that may be evaluated in this article, or claim that may be made by its manufacturer, is not guaranteed or endorsed by the publisher.
